# Phytochemical content and antioxidant activity in aqueous extracts of *Cyclocarya paliurus* leaves collected from different populations

**DOI:** 10.7717/peerj.6492

**Published:** 2019-02-19

**Authors:** Mingming Zhou, Yuan Lin, Shengzuo Fang, Yang Liu, Xulan Shang

**Affiliations:** 1 College of Forestry, Nanjing Forestry University, Nanjing, Jiangsu, China; 2 Co-Innovation Center for Sustainable Forestry in Southern China, Nanjing Forestry University, Nanjing, Jiangsu, China

**Keywords:** Flavonoid, Geographical variation, Water-soluble polysaccharide, Antioxidant activity, Polyphenol, *Cyclocarya paliurus*

## Abstract

*Cyclocarya paliurus* (Batal) Iljinskaja is a multiple function tree species, but its main utilization is for the harvesting of its leaves as materials for tea production and recently as ingredients for the food industry. In this study aqueous extracts of *C. paliurus* leaves collected from 21 natural populations were evaluated for their phytochemical content and antioxidant activity. The content of water-soluble polysaccharide, total flavonoid and total polyphenol varied from 66.05 to 153.32 mg/g, 9.01 to 19.65 mg/g and 20.80 to 52.69 mg/g, respectively. Quercetin-3-*O*-glucuronide, aemferol-3-*O*-glucuronide and 3-*O*-caffeoylquinic acid were the major phenolic components in aqueous extracts of *C. paliurus* leaves. Both redundancy analysis and Pearson's correlation analysis indicated that water-soluble polysaccharide, total polyphenol, total flavonoid, quercetin-3-*O*-glucuronide, 3-*O*-caffeoylquinic acid and 4-*O*-caffeoylquinic acid were significantly correlated with antioxidant activity, but total polyphenol showed the greatest contribution to antioxidant capacity. The antioxidant activity of the 21 populations was classified into six distinct groups based on the squared Euclidean distance. These results would provide a theoretical basis for obtaining the greatest yield of targeted antioxidant phytochemicals of *C. paliurus* leaves for tea and food ingredient production.

## Introduction

Reactive oxygen species could lead to oxidative damages of cell membrane, protein and DNA, which are associated with several chronic diseases such as inflammation, cancer, cardiovascular disease and so on (Pietta, 2000). Extensive research has demonstrated that the intake of plant product containing antioxidant compounds could reduce the risk of being attacked by numerous diseases (Xie et al., 2015). In recent years, increasing attention has been paid to phytochemicals, partly because of their multiple bioactive activities including antioxidant activity (Abrahim, Abdul-Rahman & Aminudin, 2018). It was commonly known that phenolic compounds and polysaccharides from plant resources play an important role in antioxidant activity (Kähkönen et al., 1999; Yang, Chen & Gu, 2012; Liew et al., 2018).

*Cyclocarya paliurus* (Batal) Iljinskaja, also known as “sweet tea tree” because of its sweet flavor of the leaves, is mainly distributed in highlands of sub-tropical areas of China (Fang et al., 2006). In China, its leaves have been mainly used for tea production and as a food industry ingredient (Fang et al., 2011). Pharmacological studies indicated that diverse biological activities including antioxidant, antimicrobial and antidiabetic activities were found in the extracts of *C. paliurus* leaves, which were ascribed to the synergies of abundant phytochemicals such as flavonoids, triterpenoids, polyphenolics, polysaccharides and other compounds (Xie et al., 2012; Yang et al., 2018; Liu et al., 2018a, 2018b). In the past decades, the leaves of *C. paliurus* have been commonly extracted with ethanol to analyze the contents and bioactivities of various phytochemicals (Zhang et al., 2010; Deng et al., 2015; Zhu et al., 2015; Cao et al., 2017), while less attention has been paid on its aqueous extracts. For example, choosing five geographical locations, the effects on streptozotocin-induced diabetic mice were evaluated with both ethanol and aqueous extracts of *C. paliurus* leaves (Liu et al., 2018a), while only the water-soluble polysaccharide content and antioxidant activity were studied in aqueous extracts of *C. paliurus* leaves collected at different geographic locations (Liu et al., 2018b). However, apart from polysaccharides, phenolic acid, flavonoid and triterpenoid were observed in aqueous extracts of *C. paliurus* leaves (Liu et al., 2018a), and these phytochemicals were likely to contribute to antioxidant activity in the aqueous extracts, especially phenols compounds. To our knowledge, there was little information available about the effects of these key components in the aqueous extracts on antioxidant activity. Indeed, the main utilization of *C. paliurus* leaves is to make nutraceutical tea for drinking recently, therefore it is very important to understand the contents and bioactivities of various phytochemicals in the aqueous extracts. Meanwhile, aqueous extraction was also a feasible and convenient way to obtain the bioactive compounds from plants when compared with other extraction solvents.

Many literatures confirmed that there were significant differences in phytochemical production and antioxidant activity among different geographical locations. For instance, significant differences in the content of total flavonoid and total phenolic and antioxidant capacity were reported in the extracts of *Thymus* and *Rhus* species as well as ginkgo leaves collected from different geographical regions (Itidel et al., 2013; Sati et al., 2013; Tohidi, Rahimmalek & Arzani, 2017). Variations of phenolic compounds and antioxidant activity were also observed in pyrola collected from different locations (Zhang et al., 2013). Moreover, Liu et al. (2018b) reported that water-soluble polysaccharide content and antioxidant activity were significantly different in the *C. paliurus* leaves from various natural populations.

In the previous studies, genetic variations of selected flavonoids (quercetin, kaemferol, isoquercitrin) have been reported in ethanol extracts of *C. paliurus* leaves under homogenous environmental conditions (Fang et al., 2011; Cao et al., 2018). Moreover, seven flavonoids and three phenolic acids were found to differ quantitatively in the ethanol extracts of *C. paliurus* leaves from different populations (Cao et al., 2017). To our knowledge, there was no information available on phytochemical content and antioxidant activity in aqueous extracts of *C. paliurus* leaves from the natural populations of its whole distribution areas. It was noted that a large production of *C. paliurus* leaves is required for tea and food ingredient production, especially for tea production. However, there are not enough *C. paliurus* plantations for leaf production and most of the leaves are still harvested from its natural forest. Therefore, variation of phytochemical content and antioxidant activity in aqueous extracts of *C. paliurus* leaves collected from 21 natural populations was investigated in this study. The objectives of this study were to seek for the components with a great contribution to antioxidant activity, and to screen out superior natural populations containing greatest yield of antioxidant phytochemicals in aqueous extracts of *C. paliurus* leaves. These results are not only helpful when collecting leaves for the nutraceutical tea production, but also can provide some information for understanding the relationship between phytochemicals and antioxidant activity in *C. paliurus* leaves.

## Material and methods

### Plant material

**Figure 1:**
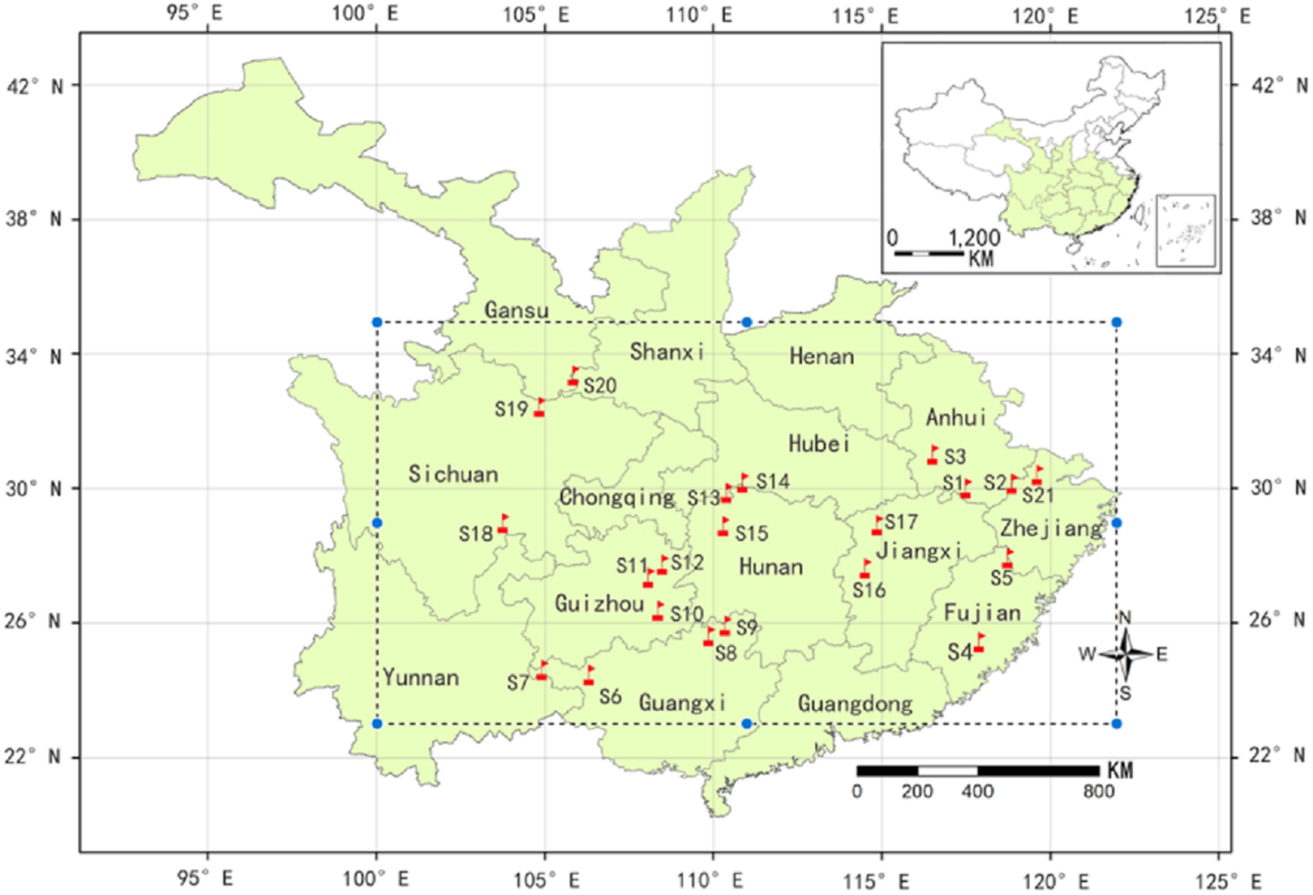
A map showing natural distribution of *C. paliurus* (dotted line box) and geographical locations of 21 populations sampled in this study (red flag). The populations codes were as follows: S1 (Guniujiang), S2 (Jixi) and S3 (Shucheng) from Anhui province; S4 (Niumulin) and S5 (Pucheng) from Fujian province; S6 (Baise), S7 (Jinzhongshan), S8 (Longsheng) and S9 (Ziyuan) from Guangxi province; S10 (Jianhe), S11 (Shiqian) and S12 (Yinjiang) from Guizhou province; S13 (Hefeng) and S14 (Wufeng) from Hubei province; S15 (Yongshun) from Hunan province; S16 (Fenyi) and S17 (Xiushui) from Jiangxi province; S18 (Muchuan), S19 (Qingchuan) from Sichuan province; S20 (Lueyang) from Shanxi province; S21(Anji) from Zhejiang province). For the detailed geographical and climatic information, please see Supplementary File (Table S1).

A total of 229 samples were collected from 21 natural populations in October 2014 throughout the major distribution areas of *C. paliurus* (Fig. 1). The longitude, latitude and altitude were measured by GPS at each sample site while the climate data of the populations was collected from Global Climate Data (http://worldclim.org/). The detailed method of sample collection and pre-treatment was described as Liu et al. (2018b). At each region, the leaves from six to 30 dominant trees were collected and mixed as a population sample. The samples were dried to a constant weight at 70 °C and then ground into fine powder. All samples were kept at room temperature before extraction.

### Sample preparation

The 30 mL of distilled water was added to 0.1 g of sample in tube to perform ultrasonic-assisted extraction. After 45 min, the solution was transferred to incubate for 15 min at 90 °C in water bath. The residues were discarded and the supernatants were retained after the solution was filtered. The obtained extracts were separated on C18 solid phase extraction column to analyze the content of water-soluble polysaccharide, total flavonoid, total polyphenol and antioxidant activity. The sample was performed in triplicate. Moreover, the extracts were also filtered through a 0.22 μm syringe filter in duplicate before high-performance liquid chromatography (HPLC) analysis.

### Measurement of water-soluble polysaccharide content

The water-soluble polysaccharide was assessed according to the phenol-sulfuric acid method (DuBois et al., 1956). Briefly, 0.05 mL of sample was diluted with distilled water to 0.5 mL and then 0.3 mL of phenol and two mL of sulfuric acid were added to the solution. The reaction solution was incubated for 30 min at room temperature. The absorbance was measured at 490 nm. The water-soluble polysaccharide content was assessed by plotting standard curve of glucose and expressed as per milligrams glucose equivalent.

### Measurement of total flavonoid and total polyphenol content

The total flavonoid content was estimated by using the aluminum trichloride colorimetric method (Li et al., 2006). Briefly, one mL of the sample was mixed with one mL of methanolic solution of AlCl_3_ (1%, w/v) and then diluted with methanol to 10 mL. The 10 mL of solution was shaken well and then incubated for 15 min at room temperature. The absorbance was measured at 415 nm. The total flavonoid content was calculated according to the standard curve of rutin and expressed as per milligrams rutin equivalent.

The total polyphenol content was determined by using the Folin–Ciocalteu (FC) colorimetric method (Xie et al., 2015). Briefly, 0.5 mL of sample was diluted with distilled water to one mL and 1.0 mL of FC reagent was added to the one mL of diluted sample. After 5 min, three mL of sodium carbonate (20%, w/v) was added to the mixture and then the reaction solution was stand for 1 h in the dark. The absorbance was measured at 765 nm. The total polyphenol content was assessed by plotting the calibration curve of gallic acid and expressed as per milligrams gallic acid equivalent.

### Measurement of individual flavonoid and phenolic acid content by HPLC

A high-performance liquid chromatography method was used to detect the flavonoid and phenolic acid content (Cao et al., 2018) with slight modifications. The mobile phases constitutions were water/acetic acid (10,000:1, v/v) (A) and acetonitrile (B). The flow rate was one mL/min and the detection wavelength was 205 nm. The column temperature was 45 °C. The gradient elution condition was from 8% (B) to 19% (B) over 13 min (from 0 to 13 min); to 21% (B) over 15 min (from 13 to 28 min); to 50% (B) over 14 min (from 28 to 42 min), to 50% (B) over 4 min (from 42 to 46 min) and re-equilibration over 10 min.

### Measurement of antioxidant activity

The 2,2'-Diphenyl-1-picrylhydrazyl (DPPH) radical scavenging ability was measured by the method of Xie et al. (2015) with minor modification. Briefly, the sample (one mL, 0.25–2.5 mg/mL) was mixed with 2.5 mL of 0.5 mM methanolic solution of DPPH. The mixture was incubated for 30 min at room temperature in the dark. Eventually, the absorbance was detected at 517 nm. The equation of percent inhibition of DPPH radical was: ((A_0_−A_1_)/A_0_) × 100, where A_0_ and A_1_ are the absorbance of the blank and samples, respectively. Ascorbic acid (VC) and butylated hydroxytoluene (BHT) were used as positive controls. Each sample of six gradients was detected in triplicate. The sample concentration at 50% inhibition of DPPH radical (IC_50_) was calculated by fitting the percent inhibition of six concentrations (0.25–2.5 mg/mL).

The 2,2-azino-bis(3-ethylbenzothiazoline-6-sulfonic acid) (ABTS) radical scavenging activity was assessed using the method of Xia et al. (2014) with minor modifications. Briefly, the mixture of equivalent volume of 7 mM ABTS and 4.9 mM potassium persulfate were incubated for 16 h at room temperature in the dark to produce ABTS^+^. Then the ABTS^+^solution was diluted with ethanol solution to an absorbance of 0.7 (± 0.02) at 734 nm. A 3.9 mL of diluted ABTS^+^ solution was added to six sample solutions of different concentrations (0.1 mL, 0.25–2.5 mg/mL), respectively. The mixture was incubated for 15 min at room temperature in the dark. The absorbance was measured at 734 nm. The percent inhibition of ABTS radical was calculated as the equation: [(A_0_−A_1_)/A_0_] × 100, where A_0_ and A_1_ are the absorbance of the blank and samples, respectively. VC and BHT were used as positive controls. Each sample of six gradients was detected in triplicate. The sample concentration at 50% inhibition of ABTS radical (IC_50_) was calculated by fitting the percent inhibition of six concentrations (0.25–2.5 mg/mL).

Reducing power was determined by the colorimetric method (Xia et al., 2014). The sample (one mL, 0.25–2.5 mg/mL) was mixed 2.5 mL of phosphate buffer (0.2 M, PH 6.6) and 2.5 mL of potassium ferricyanide (10%). The mixture was incubated for 20 min at 50 ^°^C. Then, 2.5 mL of trichloroacetic acid was added to the reaction solution. Then the 2.5 mL of solution was mixed with 2.5 mL of distilled water and 0.5 mL of ferric chloride (1%). Eventually, the absorbance was detected at 700 nm. VC and BHT were used as positive controls. Each sample of six gradients was detected in triplicate. The sample concentration at 0.5 of absorbance (EC_50_) was assessed according to linear fitting the sample absorbance of six concentrations.

### Statistical analysis

All data were expressed as mean ± standard deviation (SD). One-way analysis of variance was conducted to detect quantitative difference of the phytochemicals and antioxidant activity in *C. paliurus* leaves among different populations followed by Turkey's multiple range tests. Redundancy analysis (RDA) and Pearson's correlation analysis were performed to assess the connection of different indexes. Additionally, hierarchical cluster analysis (HCA) was conducted to classify samples from different geographic locations by using the radical scavenging capacities of DPPH and ABTS, and reducing power as three variables. All statistical analyses were performed by using SPSS 19.0 software (SPSS, Chicago, IL, USA).

## Results

### Variation in phytochemical content

**Figure 2:**
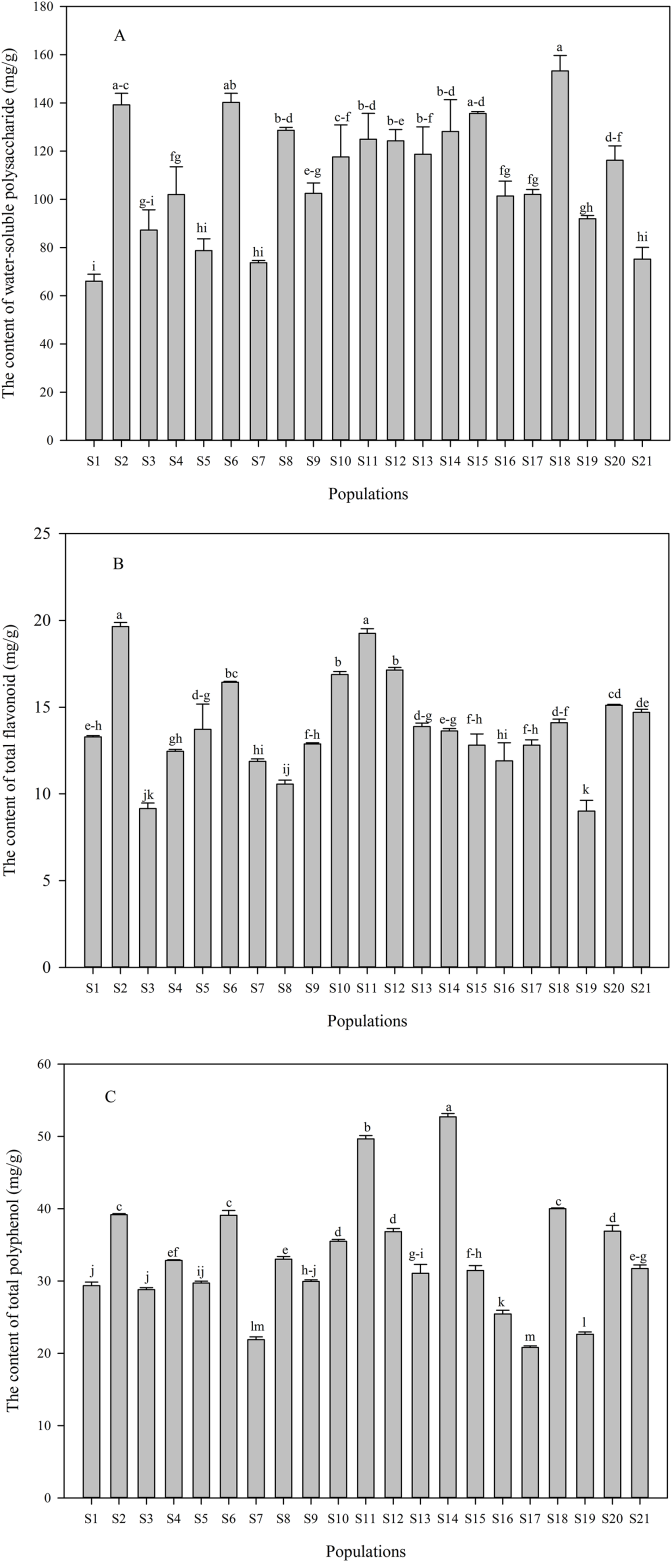
Variation in the contents of water-soluble polysaccharide (A), total flavonoid (B) and total polyphenol (C) in *C. paliurus* leaves collected from 21 populations. Data were expressed as mean ± SD of triplicate experiments. Different letters indicate significant difference for each measured index among the populations (*p* ≤ 0.05).

Contents of water-soluble polysaccharide and phenolic compounds in *C. paliurus* leaves differed significantly across different populations, and contents of water-soluble polysaccharide, total flavonoid and total polyphenol ranged from 66.05 to 153.32 mg/g (Fig. 2A), 9.01 to 19.65 mg/g (Fig. 2B) and 20.80 to 52.69 mg/g (Fig. 2C), respectively. The highest content of phenolic compounds in *C. paliurus* leaves was recorded in the S2 or S14 population, while a peak value of water-soluble polysaccharide was detected in S18 population. Quercetin-3-*O*-glucuronide (from 1.61 to 5.66 mg/g), kaemferol-3-*O*-glucuronide (from 1.80 to 4.40 mg/g) and 3-*O*-caffeoylquinic acid (from 2.51 to 5.60 mg/g) were the major phenolic components (Table 1). However, kaemferol-3-*O*-glucoside was not detected in most populations, with the range of 1.49 to 2.00 mg/g in S4, S6, S17, S18, S19 and S20 populations (Table 1).

**Table 1:** Flavonoid and phenolic acid contents in *C. paliurus* leaves from the 21 populations.

Populations	Flavonoid content (mg/g)							Phenolic acid content (mg/g)	
	Quercetin-3-*O*-glucuronide	Quercetin-3-*O*-galactoside	Isoquercitrin	Kaempferol-3-*O*-glucuronide	Kaempferol-3-*O*-glucoside	Quercetin-3-*O*-rhamnoside	Kaempferol-3-*O*-rhamnoside	3-*O*-caffeoyluinicacid	4-*O*-caffeoylquinicacid
S1	2.48 ± 0.13i–k	0.72 ± 0.02ef	0.04 ± 0.00fg	2.15 ± 0.07h-j	nd	nd	0.22 ± 0.00i–k	2.51 ± 0.05j	nd
S2	5.66 ± 0.06a	1.01 ± 0.00a	0.13 ± 0.00d	3.13 ± 0.02cd	nd	0.61 ± 0.00a	1.54 ± 0.01a	4.04 ± 0.26c–e	2.42 ± 0.18a
S3	2.84 ± 0.11g–j	0.54 ± 0.02ij	nd	2.53 ± 0.07f–h	nd	0.47 ± 0.00g	0.26 ± 0.01h-j	3.25 ± 0.07hi	1.04 ± 0.03b–e
S4	2.14 ± 0.00k	0.60 ± 0.03g–j	0.11 ± 0.01de	1.96 ± 0.18ij	1.99 ± 0.15a	0.41 ± 0.00h	0.35 ± 0.04g–i	3.29 ± 0.21g–i	0.88 ± 0.24e
S5	3.43 ± 0.03d–f	0.76 ± 0.04d–f	0.12 ± 0.01d	2.65 ± 0.01e–g	nd	0.51 ± 0.01d–g	0.32 ± 0.01hi	3.39 ± 0.00f–i	1.09 ± 0.00b–e
S6	3.11 ± 0.19f–h	0.90 ± 0.06a–c	0.20 ± 0.02c	2.49 ± 0.11f–h	1.51 ± 0.01c	0.53 ± 0.01c–f	1.00 ± 0.02d	4.70 ± 0.30b	0.96 ± 0.04c–e
S7	3.17 ± 0.12e–g	0.59 ± 0.03h-j	nd	2.72 ± 0.04e–g	nd	0.50 ± 0.01d–g	0.68 ± 0.01e	3.07 ± 0.10hi	nd
S8	2.94 ± 0.13g–i	0.74 ± 0.04ef	0.10 ± 0.02d–f	2.57 ± 0.05fg	nd	0.51 ± 0.00d–g	1.25 ± 0.02bc	3.86 ± 0.20d–f	1.02 ± 0.02b–e
S9	2.42 ± 0.04jk	0.58 ± 0.00ij	0.05 ± 0.00e–g	2.15 ± 0.22h-j	nd	0.50 ± 0.00d–g	0.08 ± 0.01kl	3.41 ± 0.01f–i	0.89 ± 0.01e
S10	3.65 ± 0.11c–e	0.71 ± 0.03e–h	0.10 ± 0.02d–f	3.55 ± 0.06b	nd	0.57 ± 0.01a–c	1.18 ± 0.02c	4.75 ± 0.23b	1.41 ± 0.06b
S11	4.01 ± 0.05bc	0.92 ± 0.02ab	0.12 ± 0.01d	3.35 ± 0.01bc	nd	nd	0.12 ± 0.01j-l	3.58 ± 0.01e–h	1.02 ± 0.00b–e
S12	3.80 ± 0.09b–d	0.87 ± 0.01b–d	0.19 ± 0.02c	2.68 ± 0.02e–g	nd	0.55 ± 0.03b–d	0.86 ± 0.02d	4.33 ± 0.03b–d	1.30 ± 0.02b–d
S13	3.93 ± 0.21bc	0.57 ± 0.06ij	0.09 ± 0.02d–f	2.46 ± 0.00f–h	nd	0.49 ± 0.03e–g	0.63 ± 0.01e	5.60 ± 0.08a	1.15 ± 0.22b–e
S14	5.29 ± 0.12a	0.82 ± 0.03b–e	0.02 ± 0.00g	4.40 ± 0.04a	nd	0.59 ± 0.01ab	1.59 ± 0.05a	4.53 ± 0.02bc	1.39 ± 0.00b
S15	2.89 ± 0.07g–j	0.55 ± 0.01ij	nd	2.35 ± 0.05g–i	nd	0.49 ± 0.01e–g	0.47 ± 0.01fg	4.06 ± 0.07c–e	1.08 ± 0.02b–e
S16	2.57 ± 0.03i–k	0.83 ± 0.02b–e	0.13 ± 0.01d	3.14 ± 0.02cd	nd	0.54 ± 0.00b–e	0.69 ± 0.00e	3.36 ± 0.03f–i	0.94 ± 0.00de
S17	2.65 ± 0.24h-j	0.79 ± 0.07c–e	0.12 ± 0.03d	3.03 ± 0.30c–e	1.49 ± 0.00c	0.53 ± 0.03c–f	0.61 ± 0.15ef	2.99 ± 0.14ij	0.92 ± 0.05de
S18	4.24 ± 0.17b	0.72 ± 0.02e–g	0.43 ± 0.03a	3.32 ± 0.06bc	1.63 ± 0.03bc	0.57 ± 0.01a–c	1.34 ± 0.02b	3.82 ± 0.07d–g	1.34 ± 0.01bc
S19	1.61 ± 0.00l	0.76 ± 0.00d–f	0.09 ± 0.00d–f	1.80 ± 0.00j	1.59 ± 0.01bc	0.54 ± 0.00c–e	0.41 ± 0.02gh	3.35 ± 0.01f–i	0.90 ± 0.03e
S20	2.70 ± 0.03g–j	0.66 ± 0.01f–i	0.35 ± 0.02b	2.79 ± 0.03d–f	1.70 ± 0.10b	0.49 ± 0.00fg	0.69 ± 0.01e	4.36 ± 0.12b–d	1.03 ± 0.21b–e
S21	3.10 ± 0.15f–h	0.52 ± 0.00j	0.07 ± 0.00d–g	2.35 ± 0.04g–i	nd	0.49 ± 0.00fg	0.07 ± 0.02l	2.88 ± 0.12ij	0.91 ± 0.04de

**Note:**

Data were expressed as mean ± SD of duplicate experiments. Different letters indicate significant difference in compounds contentin *C. paliurus* leaves from 21 population (*p* ≤ 0.05). Nd was not detected.

### Variation in antioxidant activity

**Figure 3:**
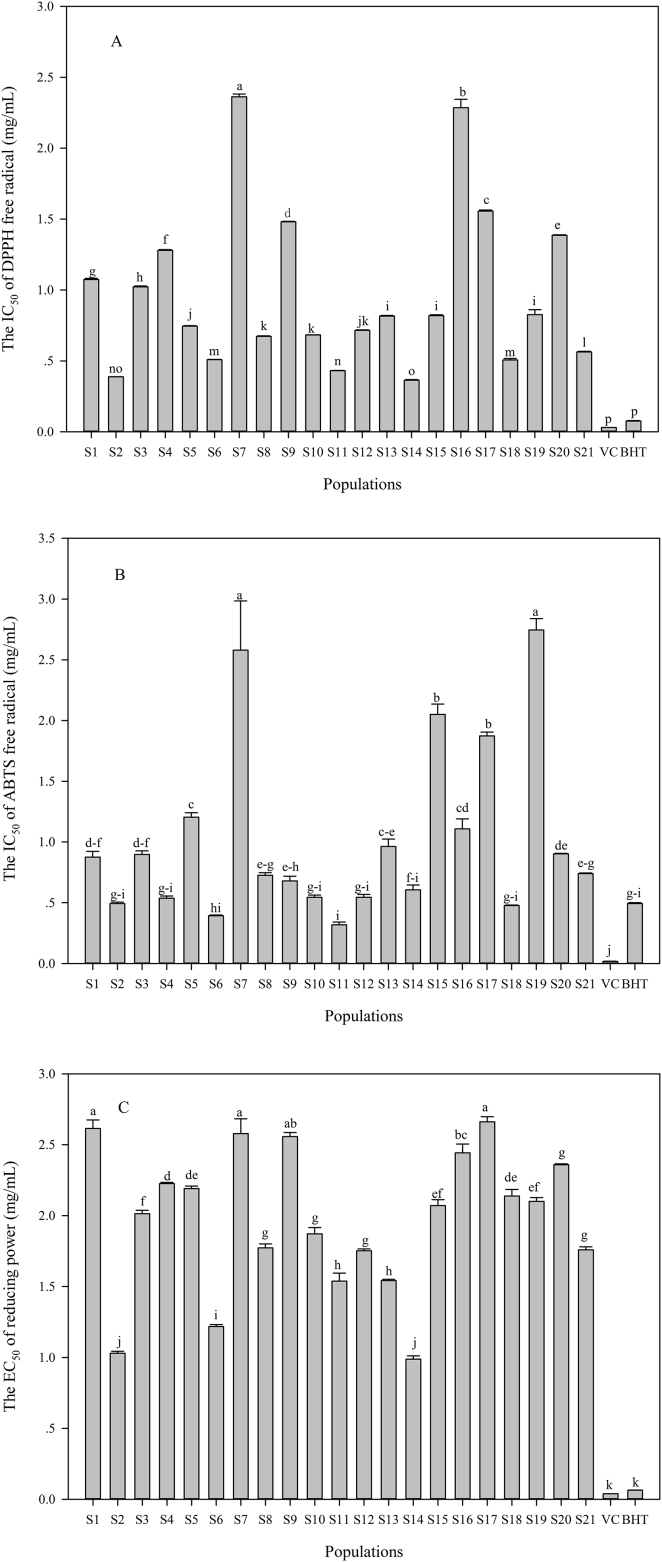
Variation in antioxidant activities (A: IC_50_ of DPPH; B: IC_50_ of ABTS; C: EC_50_ of reducing power) in the aqueous extracts of *C. paliurus* leaves collected from 21 populations. Data were expressed as mean ± SD of triplicate experiments. Different letters indicate significant difference for each antioxidant activity among the populations (*p* ≤ 0.05).

The DPPH and ABTS radical scavenging capacity, and reducing power were performed to assess antioxidant activities in the *C. paliurus* leaves. Significant differences were found in measured antioxidant activities among the 21 populations (Fig. 3). The IC_50_ of DPPH and ABTS ranged from 0.36 to 2.36 mg/mL (Fig. 3A) and from 0.32 to 2.35 mg/mL (Fig. 3B), respectively. S14 population showed the best capacity of scavenging DPPH radical, followed by S2 and S11 populations (Fig. 3A). Regarding ABTS radical scavenging capacity, these populations including S2, S4, S6, S10, S11, S12, S14 and S18 were comparable to BHT, while S7, S15, S17 and S19 populations showed poor ability (Fig. 3B). In addition, the EC_50_ values of reducing power varied from 0.99 mg/mL to 2.66 mg/mL among the populations (Fig. 3C), with the best capacity of reducing power was observed in S2 and S14 populations, while S1, S7 and S17 populations showed poor ability (Fig. 3C).

**Figure 4:**
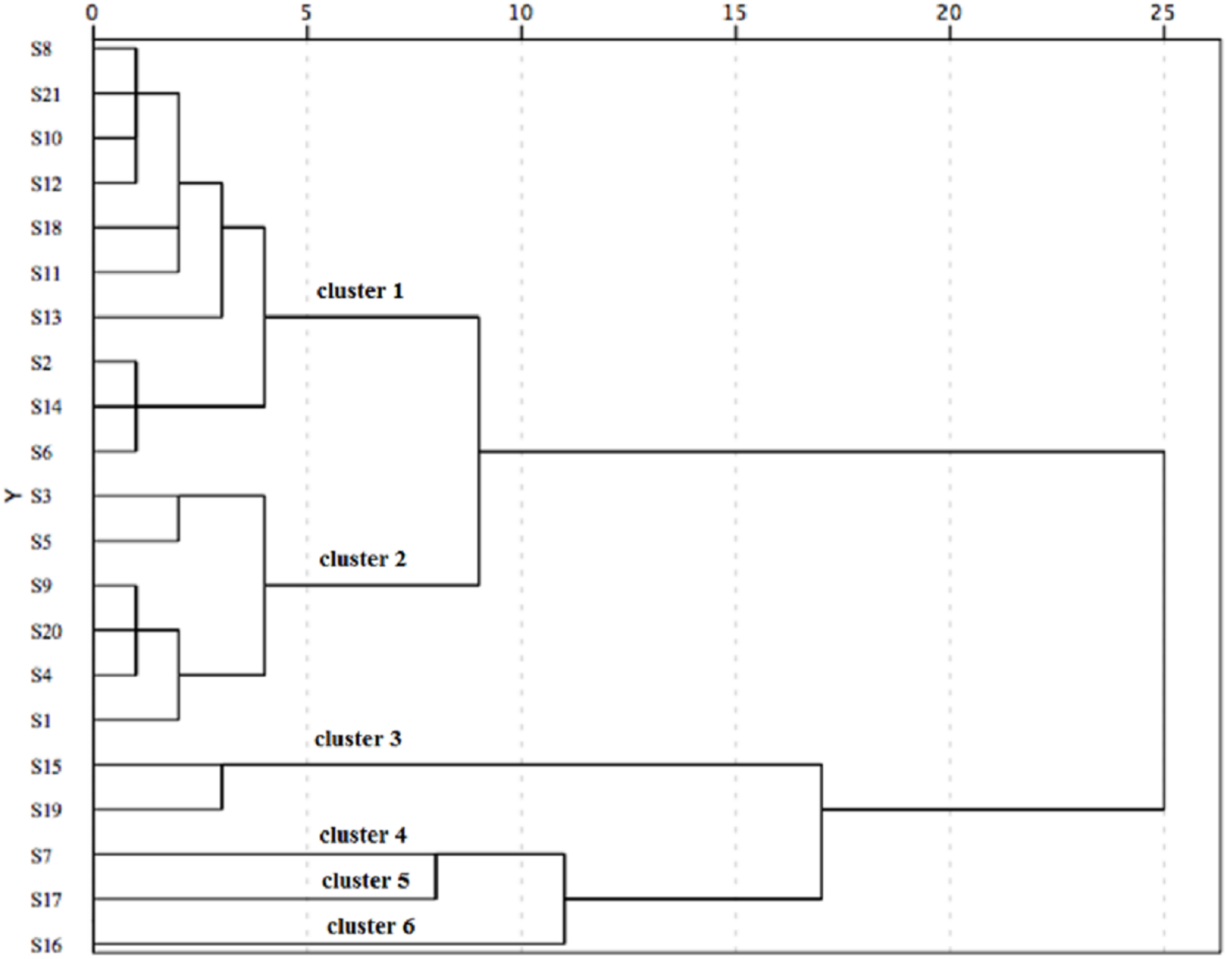
The hierarchical cluster analysis for the antioxidant activity of *C. paliurus* leaves from 21 populations based on the IC_50_ of DPPH and ABTS and EC_50_ of reducing power.

Taking the three antioxidant assays into consideration, the 21 populations were classified into six distinct groups based on the squared Euclidean distance of 5 (Fig. 4). Cluster 1 (including S2, S6, S8, S10, S11, S12, S13, S14, S18 and S21) showed good performance in antioxidant activity. Additionally, S2, S6 and S14 populations showed an excellent antioxidant activity among these populations of Cluster 1. However, Cluster 4 (S7), Cluster 5 (S17) and Cluster 6 (S16) indicated a poor capacity in antioxidant activity.

### Relationships between phytochemicals and antioxidant activity

**Figure 5:**
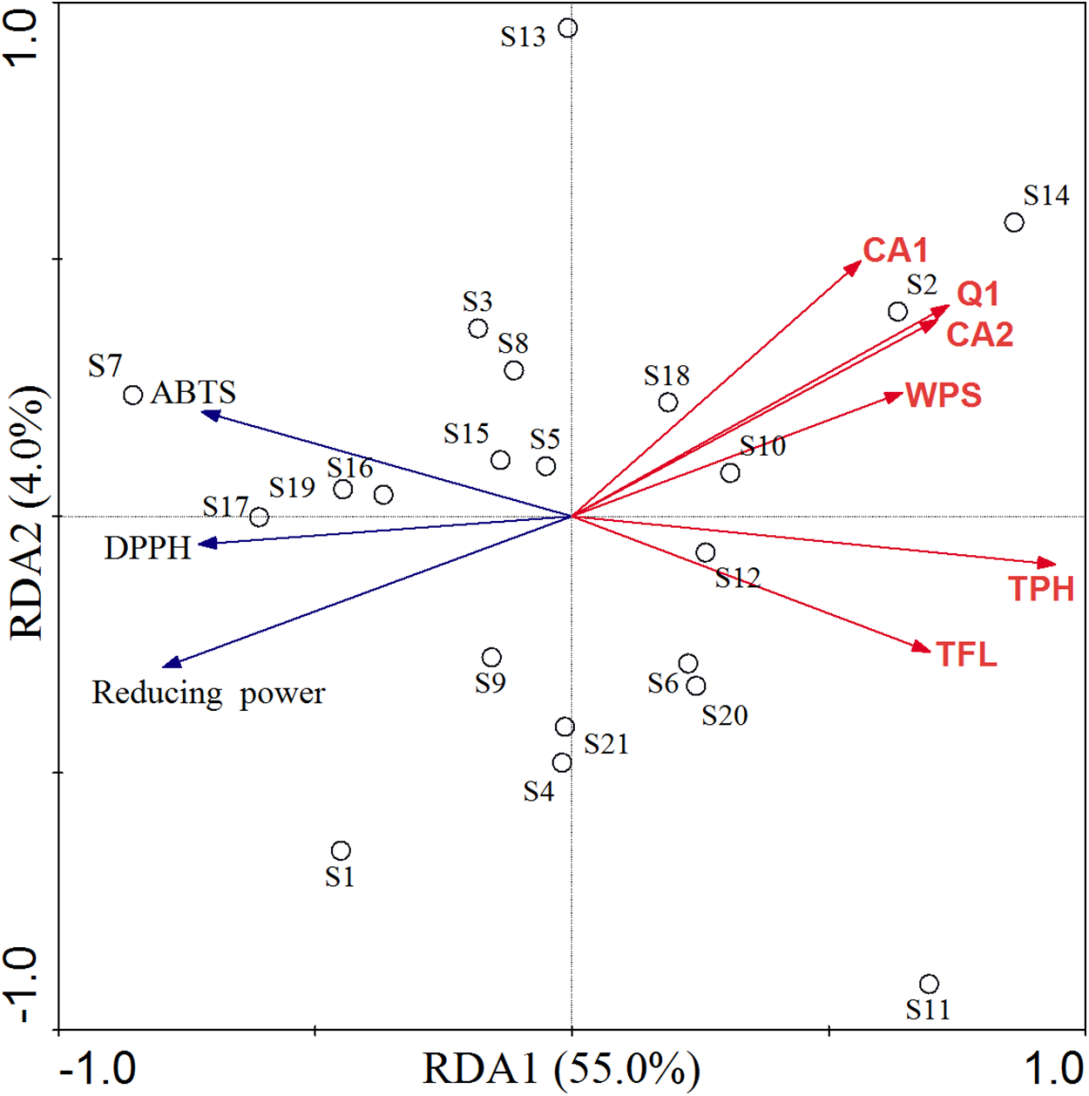
RDA of antioxidant activity constrained by contents of bioactive compounds in *C. paliurus* leaves. TFL: total flavonoid, TPH: total polyphenol, WPS: water-soluble polysaccharide, CA1: 3-*O*-caffeoylquinic acid, CA2: 4-*O*-caffeoylquinic acid, Q1: quercetin-3-*O*-glucuronide.

Redundancy analysis and Pearson's correlation analysis were carried out to determine the possible relationship between phytochemicals and antioxidant activity in *C. paliurus* leaves (Table 2; Fig. 5). The RDA model explained 59.0% of the total variation, with axis 1 and axis 2 accounted for 55.0% and 4.0%, respectively (Fig. 5). According to the permutation test, the explanatory variables retained in the model were significantly (*p* < 0.05) correlated with antioxidant activity, including total polyphenol, total flavonoid, water-soluble polysaccharide, quercetin-3-*O*-glucuronide, 3-*O*-caffeoylquinic acid and 4-*O*-caffeoylquinic acid. Based on the direction of their vectors, the IC_50_ of the DPPH and ABTS and EC_50_ of reducing power were negatively correlated with explanatory variables. In other words, all the compounds were positively correlated with antioxidant ability. Along the first axis, total polyphenol showed the greatest contribution in the ordination of indicator antioxidant, followed by quercetin-3-*O*-glucuronide, 4-*O*-caffeoylquinic acid, total flavonoid, water-soluble polysaccharide and 3-*O*-caffeoylquinic acid. It is worthy to indicate that 3-*O*-caffeoylquinic acid, 4-*O*-caffeoylquinic acid and quercetin-3-*O*-glucuronide played a strong role in the second axis.

**Table 2:** Pearson's correlation coefficients between the phytochemicals and antioxidant activity in *C. paliurus* leaves.

Index	Water-soluble polysaccharide	Total flavonoid	>Total polyphenol	3-*O*-caffeoyluinic acid	4-*O*-caffeoyluinic acid	Quercetin-3-*O*-glucuronide	Quercetin-3-*O*-galactoside	Isoquercitrin	Kaempferol-3-*O*-glucuronide	Kaempferol-3-*O*-glucoside	Quercetin-3-*O*-rhamnoside	Kaempferol-3-*O*-rhamnoside
IC_50_ of DPPH	−0.481**	−0.435**	−0.670**	−0.401*	−0.563**	−0.524**	−0.297	−0.163	−0.202	0.055	0.031	−0.305*
IC_50_ of ABTS	−0.396**	−0.565**	−0.693**	−0.317*	−0.413**	−0.457**	−0.260	−0.330*	−0.324*	0.112	0.154	−0.242
EC_50_ of reducing power	−0.558**	−0.506**	−0.725**	−0.611**	−0.661**	−0.715**	−0.442**	−0.001	−0.401**	0.189	−0.194	−0.525**

**Note:**

* and ** indicate significant effects at *p* < 0.05 and *p* < 0.01, respectively.

Pearson's correlation analysis showed that both IC_50_ and EC_50_ values were all significantly and negatively correlated with water-soluble polysaccharide, total flavonoid, total polyphenol, quercetin-3-*O*-glucuronide, 3-*O*-caffeoylquinic acid and 4-*O*-caffeoylquinic acid (Table 2). Moreover, quercetin-3-*O*-glucuronide showed the best antioxidant capacity among investigated flavonoids, while 4-*O*-caffeoyluinic acid was better between the two phenolic acids.

## Discussion

The present study proved that contents of water-soluble polysaccharide and phenolic compounds in leaves were significantly different in 21 populations of *C. paliurus*, which was consistent with previous reports (Cao et al., 2017; Liu et al., 2018a). Compared with other plant species, the contents of total flavonoid and total polyphenol in the *C. paliurus* leaves were much higher than those of aqueous extracts from the leaves of indigo plant and Tossa jute, but similar to that of ginkgo leaves (Sati et al., 2013; Heo et al., 2014; Yakoub et al., 2018). However, the total polyphenol content in *C. paliurus* leaves was lower than those of aqueous extracts from *Cyclopia intermedia*, *ugni molinae* and *Nepeta* species leaves (Dube, Meyer & Marnewick, 2017; López de Dicastillo et al., 2017; Dienaitė et al., 2018). Moreover, water-soluble polysaccharide content in the *C. paliurus* leaves was far beyond that of a previous study (Liu et al., 2018b), which may be due to the difference in the extraction method used. Our results also showed that the content of water-soluble polysaccharide was far richer than phenolic compounds in *C. paliurus* leaves, inconsistent with previous study (Liu et al., 2018a).

As indicated that there were significant differences in antioxidant activities among the 21 populations (Fig. 3). However, compared with other plant species, the IC_50_ values of DPPH in *C. paliurus* leaves were all higher than that of *Rhus* species and *Aspalathus linearis*, but lower than that of *Saraca asoca* bark and Tossa jute leaves (Itidel et al., 2013; Bhebhe, Chipurura & Muchuweti, 2015; Tewari et al., 2017; Yakoub et al., 2018). In addition, both IC_50_ values of DPPH and ABTS in the *C. paliurus* leaves were higher than those of *Pluchea indica* leaves but comparable to those of aqueous extracts from goji berry (Vongsak et al., 2018; Skenderidis et al., 2018). However, the reducing power in *C. paliurus* leaves was lower than that of *Pinus halepensis* and *Thymus* essential oils (Djerrad, Kadik & Djouahri, 2015; Tohidi, Rahimmalek & Arzani, 2017).

Genotype and environmental factors have an important influence on the production of phytochemicals and thereby influenced the antioxidant activity. Hare (2002) and Sosa et al. (2005) indicated that the phytochemical diversification among different geographical regions was mainly caused by environmental conditions, while the genotype performed slight impact on phytochemicals. Li et al. (2017) indicated the genetic diversity was low among natural *C. paliurus* populations based on the inter-simple sequence repeat and simple sequence repeat analysis. Our results found that the effects of environmental factors on various phytochemicals were different. The annual average temperature was significantly and negatively correlated with 4-*O*-caffeoylquinic acid, quercetin-3-*O*-glucuronide, DPPH radical scavenging capacity and reducing power (Table 3). Our results are consistent with the report by Zhang et al. (2013), where the high phenolic content and antioxidant capacity may result from low temperature stress. Meanwhile, annual average precipitation also showed significant and negative correlations with isoquercitrin and kaemferol-3-*O*-glucoside, supporting the point that low annual average precipitation may induce the synthesis of the particular phenolic compounds (Zhang et al., 2013). However, most phytochemicals were not significantly correlated with environmental factors investigated in our study (Table 3). The reason may be explained by the viewpoint of Lavola et al. (2017) who considered the microclimatic and topographical conditions lead to variations of phytochemicals in different regions, whereas we only collected climate data of the populations from Global Climate Data.

**Table 3:** Pearson's correlation coefficients between phytochemicals and geographic and climatic factors.

Traits	Longitude	Latitude	Altitude	Annual average precipitation	Annual sunlight	Annual averagetemperature
Water-soluble polysaccharide	−0.432**	−0.098	0.207	−0.190	−0.324**	−0.030
Total flavonoid	−0.013	−0.108	−0.005	−0.049	−0.084	−0.161
Total polyphenol	−0.169	0.016	0.045	−0.153	−0.154	−0.187
3-*O*-caffeoyluinic acid	−0.420**	−0.008	0.340*	−0.282	−0.250	−0.279
4-*O*-caffeoyluinic acid	0.108	0.211	−0.093	0.052	−0.036	−0.318*
Quercetin-3-*O*-glucuronide	0.019	0.052	0.052	0.126	−0.042	−0.338*
Quercetin-3-*O*-galactoside	−0.064	−0.081	0.155	0.030	−0.057	−0.120
Isoquercitrin	−0.396**	0.152	0.205	−0.360*	−0.251	0.215
Kaempferol-3-*O*-glucuronide	−0.146	0.016	0.096	−0.029	−0.133	−0.196
Kaempferol-3-*O*-glucoside	−0.283	0.112	0.186	−0.413**	−0.036	0.355**
Quercetin-3-*O*-rhamnoside	−0.139	0.006	0.202	−0.090	0.035	0.083
Kaempferol-3-*O*-rhamnoside	−0.284	−0.064	0.276	−0.087	−0.048	−0.046
IC_50_ of DPPH	−0.014	−0.206	−0.023	0.011	−0.162	0.312*
IC_50_ of ABTS	−0.199	0.161	0.308*	−0.229	−0.211	0.074
EC_50_ of reducing power	0.020	−0.041	−0.158	0.046	−0.045	0.428**

**Note:**

* and ** indicate significant effects at *p* < 0.05 and *p* < 0.01, respectively.

## Conclusions

Significant differences in phytochemicals and antioxidant activity were observed in aqueous extracts of *C. paliurus* leaves collected from 21 natural populations. Water-soluble polysaccharide was the major composition among investigated phytochemicals, while quercetin-3-*O*-glucuronide, kaemferol-3-*O*-glucuronide and 3-*O*-caffeoylquinic acid were major phenolic components. The contents of water-soluble polysaccharide, total polyphenol, total flavonoid, quercetin-3-*O*-glucuronide, 3-*O*-caffeoylquinic acid and 4-*O*-caffeoylquinic acid were significantly correlated with antioxidant capacity, but the total polyphenol content showed the greatest contribution to antioxidant capacity. Based on the HCA, the antioxidant activity of the 21 populations was classified into six distinct groups, and Cluster 1 (including 10 populations) is suggested for harvesting the leaves for future food and medical use. Our results provide not only a theoretical basis for harvesting *C. paliurus* leaves from the geographic locations, but also for understanding the relationships between phytochemicals and antioxidant activity in plants.

## Supplemental Information

10.7717/peerj.6492/supp-1 Supplemental Information 1 Raw data.

10.7717/peerj.6492/supp-2 Supplemental Information 2 Geographical and climatic information of *C. paliurus* leaves from 21 populations.

10.7717/peerj.6492/supp-3 Supplemental Information 3 HPLC chromatograms of the representative sample solution (top) and the responding standard solution containing the nine quantitative compounds (bottom). 1: 3-*O*-caffeoyluinic acid; 2: 4-*O*-caffeoyluinic acid; 3: quercetin-3-*O*-glucuronide; 4: quercetin-3-*O*-galactoside; 5: isoquercitrin; 6: kaempferol-3-*O*-glucuronide; 7: kaempferol-3-*O*-glucoside; 8: quercetin-3-*O*-rhamnoside; 9: 4,5-di-*O*-caffeoyluinic acid; 10: kaempferol-3-*O*-rhamnoside.

## Additional Information and Declarations

### Data Availability

The following information was supplied regarding data availability: Raw data are available in a Supplemental File.
