# Supplementary material for: Phytochemical content and antioxidant activity in aqueous extracts of Cyclocarya paliurus leaves collected from different populations
Source: PeerJ. 2019 Feb 19;7:e6492. doi: 10.7717/peerj.6492 (PMC6385679; doi:10.7717/peerj.6492)
Supplement: Supplemental Information 2 [file peerj-07-6492-s002.docx]

| Populations | Province | County or location | Longitude (E) | Latitude (N) | Altitude (m) | Annual average  precipitation (mm) | Annual sunlight (h) | Annual average  temperature (C) |
| --- | --- | --- | --- | --- | --- | --- | --- | --- |
| S1 | Anhui | Guniujiang | 117°31′48″ | 30°1′12″ | 399.6 | 1775 | 1800 | 13.9 |
| S2 | Anhui | Jixi | 118°27′00″ | 30°13′48″ | 752.5 | 1726 | 1926 | 12.1 |
| S3 | Anhui | Shucheng | 116°32′24″ | 31°1′12″ | 769.3 | 1606 | 1969 | 12.3 |
| S4 | Fujian | Niumulin | 117°55′48″ | 25°25′48″ | 477.0 | 1593 | 1950 | 18.1 |
| S5 | Fujian | Pucheng | 118°45′36″ | 27°55′48″ | 931.7 | 1998 | 1738 | 15.8 |
| S6 | Guangxi | Baise | 106°20′24″ | 24°27′36″ | 1478.0 | 1364 | 1906 | 15.6 |
| S7 | Guangxi | Jinzhongshan | 104°57′00″ | 24°36′36″ | 1773.1 | 1212 | 1475 | 16.7 |
| S8 | Guangxi | Longsheng | 109°53′24″ | 25°37′12″ | 1307.0 | 1629 | 1544 | 14.5 |
| S9 | Guangxi | Ziyuan | 110°22′48″ | 25°55′12″ | 842.0 | 1580 | 1308 | 15.5 |
| S10 | Guizhou | Jianhe | 108°22′48″ | 26°22′12″ | 1156.0 | 1265 | 1236 | 15.1 |
| S11 | Guizhou | Shiqian | 108°6′36″ | 27°21′00″ | 1239.0 | 1256 | 966 | 13.3 |
| S12 | Guizhou | Yinjiang | 108°30′36″ | 27°44′24″ | 1016.1 | 1231 | 1100 | 14.5 |
| S13 | Hubei | Hefeng | 110°25′12″ | 29°52′48″ | 1130.9 | 1499 | 1342 | 11.3 |
| S14 | Hubei | Wufeng | 110°54′00″ | 30°11′24″ | 969.2 | 1377 | 1533 | 12.6 |
| S15 | Hunan | Yongshun | 110°19′48″ | 28°52′48″ | 673.0 | 1492 | 1266 | 14.8 |
| S16 | Jiangxi | Fenyi | 114°31′48″ | 27°37′48″ | 558.1 | 1691 | 1737 | 15.3 |
| S17 | Jiangxi | Xiushui | 114°53′24″ | 28°55′12″ | 825.2 | 1655 | 1600 | 13.5 |
| S18 | Sichuan | Muchuan | 103°46′48″ | 28°58′12″ | 1153.1 | 1332 | 965 | 17.3 |
| S19 | Sichuan | Qingchuan | 104°51′36″ | 32°25′12″ | 1588.1 | 795 | 900 | 14.1 |
| S20 | Shanxi | Lueyang | 105°52′12″ | 33°21′36″ | 1218.2 | 714 | 1526 | 13.0 |
| S21 | Zhejiang | Anji | 119°38′24″ | 30°24′36″ | 571.8 | 1472 | 1849 | 13.4 |
