# Supplementary figures and images for: Phytochemical content and antioxidant activity in aqueous extracts of Cyclocarya paliurus leaves collected from different populations
Source: PeerJ. 2019 Feb 19;7:e6492. doi: 10.7717/peerj.6492 (PMC6385679; doi:10.7717/peerj.6492)

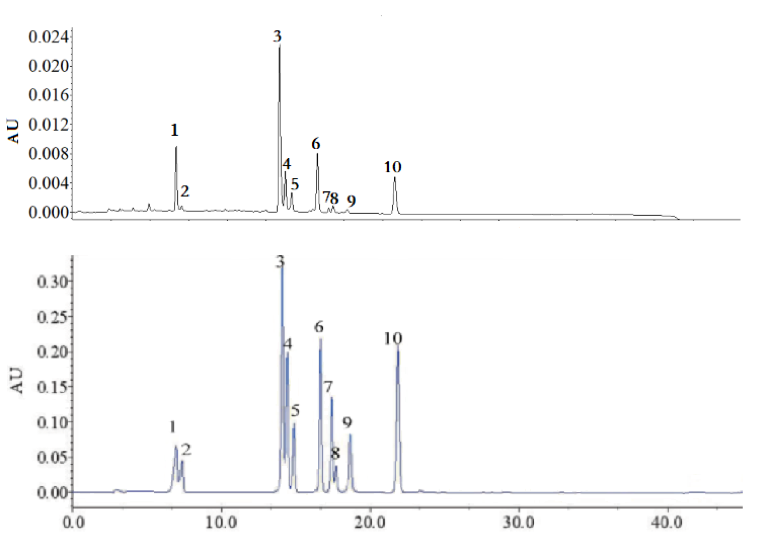

Supplement: Supplemental Information 3 — 1: 3-O-caffeoyluinic acid; 2: 4-O-caffeoyluinic acid; 3: quercetin-3-O-glucuronide; 4: quercetin-3-O-galactoside; 5: isoquercitrin; 6: kaempferol-3-O-glucuronide; 7: kaempferol-3-O-glucoside; 8: quercetin-3-O-rhamnoside; 9: 4,5-di-O-caffeoyluinic acid; 10: kaempferol-3-O-rhamnoside. [file peerj-07-6492-s003.docx]
